# Smoothness preserving layout for dynamic labels by hybrid optimization

**DOI:** 10.1007/s41095-021-0231-y

**Published:** 2021-10-27

**Authors:** Yu He, Guo-Dong Zhao, Song-Hai Zhang

**Affiliations:** 1 Department of Computer Science and Technology, Tsinghua University, Beijing, China; 2 Department of Computer Science and Technology, Tianjin University of Technology, Tianjin, China

**Keywords:** label layout, smoothness preserving, dynamic label, forced based, static optimization, hybrid optimization

## Abstract

Stable label movement and smooth label trajectory are critical for effective information understanding. Sudden label changes cannot be avoided by whatever forced directed methods due to the unreliability of resultant force or global optimization methods due to the complex trade-off on the different aspects. To solve this problem, we proposed a hybrid optimization method by taking advantages of the merits of both approaches. We first detect the spatial-temporal intersection regions from whole trajectories of the features, and initialize the layout by optimization in decreasing order by the number of the involved features. The label movements between the spatial-temporal intersection regions are determined by force directed methods. To cope with some features with high speed relative to neighbors, we introduced a force from future, called temporal force, so that the labels of related features can elude ahead of time and retain smooth movements. We also proposed a strategy by optimizing the label layout to predict the trajectories of features so that such global optimization method can be applied to streaming data.

**Electronic Supplementary Material:**

Supplementary material is available in the online version of this article at 10.1007/s41095-021-0231-y.

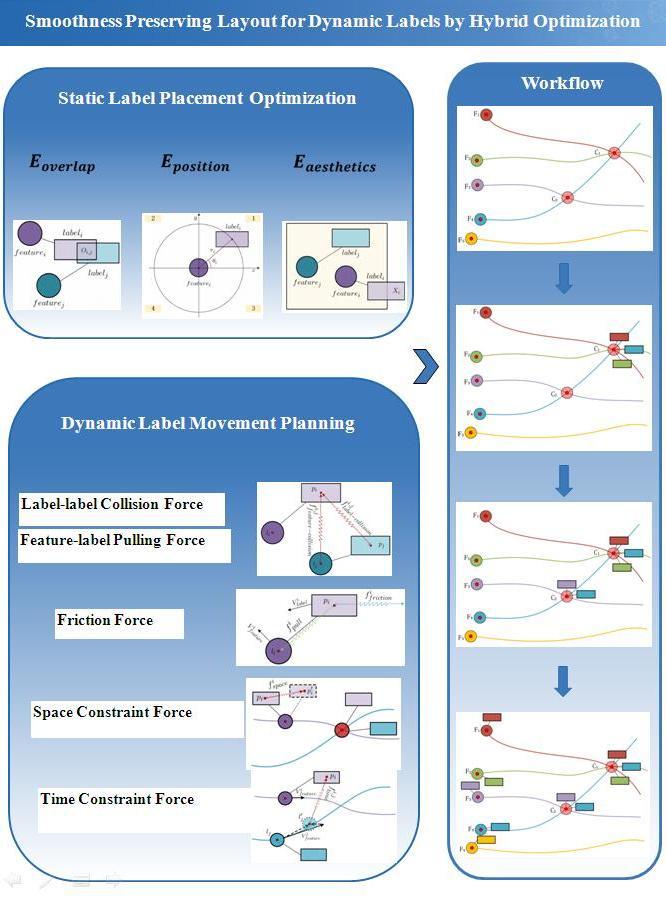

## 1 Introduction

The automated placement of labels on maps and other visualizations is a long-standing goal of information visualization. Human map makers and designers can solve the labeling problem quite well, but an automated approach is often required for large dynamic data sets of visualizations.

Most optimization problems related to label placement are NP-hard, but several heuristics with different objectives and underlying principles have been proposed over the years. In this paper, we focus on the problem of boundary labeling, that is, the labels are placed around the perimeter of the visualization and connected to their corresponding features (e.g., points or image features) with leaders (i.e., lines). A drawback of boundary labeling is that the associations between features and their labels are not immediately apparent nor easy to follow: one has to trace the leader across the visualization to find the corresponding label. In the literature, automated dynamic label placement is often split into two main approaches, each of which has its features and drawbacks:

- Global optimization with previous frames. The global optimization originally used into static label layout problem. Several aspects are taken into consideration, including relative position to its features, non-overlapping to other features, and other aesthetic rules, assuring a balanced layout without overlapping after optimization. For dynamic labeling, a common extension is optimization incorporating the label positions in previous frames. This strategy is global optimization only in spatial and NOT in temporal domain, so as to be in a dilemma in optimization occasionally. A more sophisticated approach is optimization in both spatial and temporal domain so that labels can adjust their trajectories ahead of time to remain moving smoothly. However, the problem is quite easy to formulate and quite difficult to optimize for all labels at all frames together. Moreover due to the trade off between

1 Department of Computer Science and Technology, Tsinghua University, Beijing, China. E-mail: Y. He, hooyeeevan@tsinghua.edu.cn; S.-H. Zhang, shz@tsinghua.edu.cn (✉).

2 Department of Computer Science and Technology, Tianjin University of Technology, Tianjin, China. E-mail: z15942612415@163.com.

Manuscript received: 2021-02-17; accepted: 2021-03-27

temporal coherence and all other aspects in optimization neither cannot guarantee temporal coherence especially in consecutive frames.

- Forced directed methods. All labels are moving according to the resultant force caused by all neighboring features and labels. Due to the forces calculated inverse to their relative distances, forced directed methods are inherently retaining smoothness of label movements. However, the resultant force only considers neighboring situations, and may cause interleaving and sudden changes if the labels have not been carefully places initially.

In this paper, we propose a novel approach for solving the labeling problem in hybrid optimization. We define joint set as the subset of the features who influence the label layout of others in the set. We extract the joint sets from spatial and temporal domain, and layout relative labels with global optimization in descending order of the complexity of the joint sets. Inbetween two joint sets, the force directed method is applied with initial positions of two joint sets. And thus we achieve the good initial layout on key regions and key frames, and move the labels according to resultant forces to achieve smoothness of the trajectories inbetween. In force directed framework, we also introduce a temporal force that copes with features with high speed relative to neighbors. The temporal force is calculated by the position and speed and added to the resultant forces so that the labels can elude ahead of time and retain smooth movements.

We also proposed a strategy to extend our hybrid framework to streaming data. By prediction of all feature trajectories, we estimate the joint sets in the future, so that the current layout can be optimized globally and our hybrid framework can be applied for the current layout.

Our main contributions are:

- A hybrid optimization method for dynamic label layout is proposed by cluster layout propagation to achieve smooth changing results.
- A temporal force is introduced to cope with features with high speed relative to neighbors so that the labels can elude ahead of time and retain smooth movements.
- The proposed layout method is extended to streaming data by prediction to avoid sudden changes.

## 2 Related work

Automatic label layout has been an active research area for decades, so a comprehensive review of all related work is beyond the scope of this paper. We focus on highlighting major developments and the works closely related to our approach. We refer the reader to the bibliography by Wolff and Strijk [1] for an extensive list of published heuristics and to the survey by Van Garderen [2] for a detailed literature review.

The labeling problem can be applied to geographical maps, technical maps, or any other kind of diagrams, such as 3D scene, graph, etc. Labels are not necessarily textual labels; they can also be graphical representations of any form. The label can be placed internally or adjacent to its feature sometimes including leaders. Such problem has been studied extensively. It is formulated as an optimization with several guidelines for "good" label placement [3] and non-overlapping constraints, which is known as an NP-Hard problem. Thus many approximations and heuristic approaches are proposed including integer linear programs [4], expert systems [5], simulated annealing [6], and combined optimization [7]. In recent years, Meng et al. [8] introduced a clutter model to prevent visual clutter in the layout. Lhuillier et al. [9] proposed a versatile density-based approach to label placement by applying kernel density estimation to the input features. These approaches presented a well organized layout result in the global scale, and however those optimization approaches did not take dynamic layout into consideration. In this paper, we proposed a hybrid solution taking the both advantages of global optimization [6] on global arrangement and forced based approaches on local smoothing together.

A common extension of the static label layout into dynamic labels is global optimization not only in spatial domain but also in temporal domain. However, the problem is quite easy to formulate and quite difficult to optimize for all labels at all frames together. A reasonable solution is optimization frame by frame with the initial position of labels in previous frame [10–12]. As the temporal coherence is only one aspect of all constraints, the trade off between temporal coherence and all other aspects in optimization cannot guarantee temporal coherence especially in consecutive frames.

Force-based approach is another branch for label layout. A force-based approach was first presented by Hirsch [13]. He uses a gradient-driven heuristic to label point features. Labels are placed on a circle around their corresponding features. To resolve conflicts, vectors between overlapping labels are computed based on the intersection area. As a single label can collide with multiple other annotations, the sum of all its vectors guides its movement, thus improving the global labeling layout. Attractive forces pull labels to their point feature, while repelling forces push labels away from other features. Thus, step by step, the map layout converges towards a final labeling state through a gradient descent method. As far as we know, the forces are mainly consider the influence within spatial domain. In this paper, we proposed a temporal force which can influence over time, so as to cope with features with high speed relative to neighbors, so that the labels can elude ahead of time and retain smooth movements.

Force-based approaches [14–16] are inherently preserve local smoothness for dynamic label movements when the forces are defined as smooth function. So that force-based approaches can be directly extended to dynamic label layout with temporal coherence enforcement. However, the resultant force only considers neighboring situations, and may cause interleaving and sudden changes if the labels have not been carefully places initially. To fully use the feature of local smoothness, and cope with the sudden change problem of force-based methods, we incorporated global optimization to achieve good initial place of labels. A hybrid approach is presented by Stadler et al. [17]. They obtain an initial placement with the help of image processing. Then, iterative forces improve the chosen labeling positions.

## 3 Spatiotemporal hybrid optimization

Our method focuses on high-density and high-dynamic data visualization. Taking the point-feature label placement (PFLP) as an example [6], we solve both the problem of static layout optimization and dynamic movement smoothing of labels by spatiotemporal decoupling. Two key frameworks are performed in our method: global spatiotemporal joint optimization and label motion planning based on force traction.

### 3.1 Global spatiotemporal joint optimization

In the process of this framework, we first locate all joint sets from spatial and temporal as is shown in Fig. 1(a), and sort them in descending order of the complexity. There are two joint sets $\{C_1, C_2\}$ in this example. Then we perform static label placement on the frame where the joint sets are located, and the position constraints of labels transmit according to the order of the joint sets. Finally, the initial label layout of the first frame and the label position constraint of each joint set are obtained. The entire workflow is illustrated in Fig. 1.

#### *3.1.1 Static label placement optimization*

According to the definition of the PFLP problem in Ref. [6], one or zero of the candidate positions for placing a label have to be chosen for each feature, such that the value of the objective function of the resulting labeling is minimal. Following the labeling quality rules proposed in Ref. [18], our optimization objective function is as follows:

$$E_{\text{static}} = E_{\text{overlap}} + E_{\text{position}} + E_{\text{aesthetics}}$$

which consists three energy terms: overlap between labels and labels and overlap between labels and features $E_{\text{overlap}}$, the relative position of a label to a

**Fig. 1** A workflow of global spatiotemporal joint optimization on non-real-time data.

feature $E_{\text{position}}$, and aesthetics factors $E_{\text{aesthetics}}$.

$$E_{\text{overlap}} = \sum_{i}^{N}\sum_{j \neq i}^{N} W_{\text{label}-\text{label}} O_{i,j} + \sum_{i}^{N}\sum_{j}^{N} W_{\text{label}-\text{feature}} P_{i,j}$$

where $N$ is the number of feature. As is shown in Fig. 2, $O_{i,j}$ is the area of overlap between label $i$ and label $j$, and $P_{i,j}$ is the area of overlap between label $i$ and feature $j$. $W_{\text{label}-\text{label}}$ and $W_{\text{label}-\text{feature}}$ are the corresponding weights.

$$E_{\text{position}} = \sum_{i}^{N} W_{\text{orient}} \Delta(\theta_i) + \sum_{i}^{N} W_{\text{distance}} r_i$$

where $\theta_i$ is the angle of the label in the corresponding feature-centered polar coordinate system as shown in Fig. 3(a). $\Delta(\theta_i)$ is to determine which quadrant the label is in. $W_{\text{orient}}$ has four values representing the priorities of the different quadrants. $r_i$ is the distance between the label and feature, and $W_{\text{distance}}$ is the corresponding weight.

$$E_{\text{aesthetics}} = \sum_{i}^{N} W_{\text{out-of-axes}} X_i + \sum_{i}^{N} W_{\text{intersect}} I_i$$

where $X_i$ is the area of the label out of the axes as shown in Fig. 3(b), and $I_i$ is the number of leader line intersections. $W_{\text{out-of-axes}}$ and $W_{\text{intersect}}$ are the

**Fig. 2** Two types of overlap: (a) overlap between labels and labels; (b) overlap between labels and features.

**Fig. 3** (a) Feature-centered polar coordinate system; (b) label out of the axes.

corresponding weights.

Since PFLP is NP-complete, we adopt the heuristics search to find good solutions in acceptable time. Based on the algorithm evaluation in Ref. [18], simulated annealing algorithm is applied to solve the objective function. The annealing schedule is chosen according to Ref. [6].

#### *3.1.2 Position constraint transmit*

Figure 1 shows an example to illustrate how the label position constraint transmits. Five features ($F_1$, $F_2$, $F_3$, $F_4$, $F_5$) and their corresponding motion paths are marked in different colors (red, green, purple, blue, yellow, respectively). There are two joint sets ($C_1\{F_1, F_2, F_4\}$, $C_2\{F_3, F_4\}$) in the entire spatial and temporal domain.

For the maximum joint set, we use the objective function $E_{\text{static}}$ which is proposed in Section 3.1.1 to layout the labels, and then we transmit this layout as position constraint to the next joint set based on the complexity descending order. The objective function is formulated as follows:

$$E = E_{\text{static}} + E_{\text{constraint}}$$

where $E_{\text{static}}$ is the static label placement energy term, and $E_{\text{constraint}}$ is the label position constraint transmitted from last joint set, which is formulated as

$$E_{\text{constraint}} = W_{\text{radius}} \|r_{\text{p}} - r_{\text{l}}\| + W_{\text{angle}} \|\theta_{\text{p}} - \theta_{\text{l}}\|$$

where $(r, \theta)$ is the coordinate value of the label in the corresponding feature-centered polar coordinate system as is shown in Fig. 3(a). $(r_{\text{p}}, \theta_{\text{p}})$ represents the current joint set, and $(r_{\text{l}}, \theta_{\text{l}})$ represents the last joint set. We only calculate the features which occur in both joint sets. In the above-mentioned example in Fig. 1, only $F_2$ and $F_4$ need to calculate this term in the second joint set $C_2$. After traversing all joint sets, every feature in joint sets will obtain a position constrain at the first frame. As for features where no path intersection occurs such as $F_5$, a specified initial label position will be assigned.

### 3.2 Dynamic label movement planning

In this framework, we focus on the movement of the labels, and both smoothness and readability are considered. After we obtain the initial layout constraints on key regions and key frames (Section 3.1), the force directed method is adopted to move the labels according to resultant forces to achieve smoothness of the trajectories inbetween [19]. In addition, to further improve readability, we also introduce a temporal force that copes with features with high speed relative to neighbors.

### *3.2.1 Force-based label movement*

In this paper, we adopt a free annotation type to display label placement [20]. A free label $i$ is positioned by an optional offset from its corresponding feature $i$ as illustrated in Fig. 3(a). Following the force descriptions in Ref. [19], we define four basic forces to maintain the smoothness and readability of label movement as is shown in Fig. 4.

**Label–label collision force:** To avoid overlap between labels, the first force introduced is repulsive force between labels. Since all labels are rectangles oriented in the same axis, we simplify the calculation of the distance between label $i$ and label $j$:

$$d^{i,j}_{\text{label}-\text{label}} = \max\left(|p_i - p_j| - \frac{1}{2}(s_i + s_j), 0\right)$$

where $p$ is the center position of a label and $s$ is the extent of a label. A label–label collision happens when $d_{\text{label}-\text{label}}$ is less than 0. The formula of label–label collision force is

$$f^{i,j}_{\text{label}-\text{collision}} = \min\left(\frac{d^{i,j}_{\text{label}-\text{label}}}{D_{\text{label}-\text{collision}}} - 1, 0\right)\frac{p_i - p_j}{\|p_i - p_j\|}$$

**Fig. 4** Basic forces: (a) repulsive forces between labels and repulsive forces between labels and features; (b) pulling forces between labels and the corresponding features and the friction force.

where $D_{\text{label}-\text{collision}}$ is maximum distance the force will act; an illustration of this force can be seen in Fig. 4(a).

**Label–feature collision force:** In this paper, each point feature has a specific size. Hence, repulsive forces between labels and features are also needed to prevent them from collision. Similar to label–label collision force, the distance between label $i$ and feature $j$ is

$$d^{i,j}_{\text{label}-\text{feature}} = \max\left(|p_i - l_j| - \frac{1}{2}(s_i + r_j)\right)$$

where $l$ is the center position of a feature and $r$ is the extent of a feature. The formula of label–feature collision force is as follows:

$$f^{i,j}_{\text{feature}-\text{collision}} = \min\left(\frac{d^{i,j}_{\text{label}-\text{feature}}}{D_{\text{feature}-\text{collision}}} - 1, 0\right)\frac{p_i - l_j}{\|p_i - l_j\|}$$

where $D_{\text{feature}-\text{collision}}$ is maximum distance the force will act.

**Feature–label pulling force:** To ensure the label stay close to the corresponding feature, a pulling force is needed. As is shown in Fig. 4(b), this force proportional to the distance, which is formulated as follows:

$$f^i_{\text{pull}} = -\ln\left(\left\| |p_i - l_i| - \frac{1}{2}(s_i + r_i)\right\| - D_{\text{pull}} + 1\right)\frac{p_i - l_i}{\|p_i - l_i\|}$$

where $D_{\text{pull}}$ is the minimum distance at which the force will be acted. The force acts only if

$$\left\| |p_i - l_i| - \frac{1}{2}(s_i + r_i)\right\| - D_{\text{pull}} > 0$$

**Friction force:** To stabilize the movement of the labels, a friction force is acted to each label which is formulated as

$$f^i_{\text{friction}} = -c_{\text{friction}} \cdot (V^i_{\text{label}} - V^i_{\text{feature}})$$

where $c_{\text{friction}}$ is the friction coefficient, and $V_{\text{label}}$ and $V_{\text{feature}}$ are the velocities of labels and features respectively. This force has a dampening effect on the labels except when they are moving in the same direction as their corresponding features.

### *3.2.2 Spatiotemporal force constraint*

In allusion to high density and hybrid high dynamics data, two more forces are introduced to enhance the visualization performance.

**Space constraint force:** To reduce the label movement chaos caused by the intersections of feature motion paths, a space constraint force is acted to plan the movement path of a label as shown in Fig. 5. The formulation of this force is as follows:

$$f^i_{\text{space}} = \ln\left(\|p_i - p'_i\| + 1\right) \frac{p_i - p'_i}{\|p_i - p'_i\|}$$

where $p'$ is the constraint position of label at upcoming feature joint set which is described in Section 3.1. If there is no new upcoming intersection, the constraint will remain the same as the previous one or the original one.

**Time constraint force:** The features of hybrid dynamics data have very different velocities. The basic forces mentioned in Section 3.2.1 are unable to deal with the overlap under such a large velocity difference. Therefore, we introduce a time constraint force which is acted from where the features will be in the future. As is shown in Fig. 6, $V^j_{\text{feature}}$ is much bigger than $V^i_{\text{feature}}$. $l'_j$ is the relative future position of the feature $j$, which is calculated as follows:

$$l'_i = l_i + (V^i_{\text{feature}} - V^j_{\text{feature}}) \cdot \delta t$$

where $\delta t$ is the time of the feature movement to the future. The time constraint force is formulated as

$$\begin{aligned} f^{i,j}_{\text{time}} &= \ln\left(\frac{\max(|v_i|, |v_j|)}{\min(|v_i|, |v_j|)}\right) \\ &\cdot \min\left(\frac{\|p_i - l_j\|}{\left\|p_i - l'_j\right\|} - 1, 0\right) \frac{p_i - l'_j}{\left\|p_i - l'_j\right\|} \end{aligned}$$

*3.2.3 Force combination*

After all forces are calculated for all labels, we combine them into one resultant force acting on each label. To weigh the forces against each other, we introduce a constant scaling factor for each force. The summarized force for label $i$ is

$$\begin{aligned} & f^i_{\text{resultant}} \\ &= \sum_{j \neq i} \left( w_{\text{label-collision}} f^{i,j}_{\text{label-collision}} + w_{\text{time}} f^{i,j}_{\text{time}} \right) \\ &\quad + \sum_{j} \left( w_{\text{feature-collision}} f^{i,j}_{\text{feature-collision}} \right) \\ &\quad + w_{\text{pull}} f^i_{\text{pull}} + w_{\text{friction}} f^i_{\text{friction}} + w_{\text{space}} f^i_{\text{space}} \end{aligned}$$

We introduce a virtual mass $m_i$ for label $i$. The current acceleration $a_i$ is computed by Newton's second law:

$$a_i = f^i_{\text{resultant}} / m_i$$

The time interval between successive frames is $\Delta t$, and the new velocity $v'_i$ can be calculated by Euler's integration method:

$$v'_i = v_i + a_i \cdot \Delta t$$

In this paper, we take virtual mass of labels equal to 1.

Fig. 5 Space force.

Fig. 6 Time force.

## 4 Implementation

We make two different implementations to evaluate the performance of our dynamic label layout method on both non-real-time and real-time data. They are both written in Python, and are only based on CPU calculations. Both of them use epidemiological data on COVID-19 in China. However, the first implementation used the data as recorded video, and the second one used the data as real-time video stream. In order to better visualize the epidemic data, we make several improvements to the visual elements. As is shown in Fig. 7, we extend the point feature to a ring feature to convey more information about the epidemic. We encode the cumulative number of confirmed cases of a province as the outer radius of the feature (red line in Fig. 7). The red–green ratio of the ring represents the ratio of the cumulative number of deaths to the cumulative number of cures.

Fig. 7 Feature design for epidemic data.

In addition, with so many provinces to display, the total area of all labels and features will be larger than the screen. We have to selectively hide some features and labels. Thus, we introduce a feature priority evaluation system for choosing which labels to be hidden, and the priority of a feature can be calculated as follows:

$$P^i_{\text{feature}} = \omega_1 \cdot R^i_{\text{death}} + \omega_2 \cdot R^i_{\text{cure}} + \omega_3 \cdot \ln\left(N^i_{\text{confirmed}}\right)$$

where $R_{\text{death}}$ is the ratio of death, $R_{\text{cure}}$ is the ratio of cure, $N_{\text{confirmed}}$ is the cumulative number of confirmed cases, and $\omega$ is the corresponding weight which allows the user to control tendency of priority determination. The lower priority labels or features will be hidden when overlapping with higher priority labels, but if features with different priorities are overlapping, the lower priority features do not need to be hidden. Video result demonstrations of the implementations can be found in the Electronic Supplementary Material (ESM).

### 4.1 Non-real-time data

The first implementation follows the steps described in Section 3. In the first step, we calculate the initial label positions and the position constraints at joint sets. In the second step, the priority of features is calculated to determine whether labels and features need to be hidden or not. In the third step, we calculate the resultant force for each label to guide the movement. As described in Section 3.2.3, there are six forces acting on each label. However, after feature priority is introduced, we hope that the movement of the labels with higher priority is more pronounced, and the lower priority labels will give way to the higher priority labels during movement. When two labels are displayed and $label_i$ has a higher priority than $label_j$, the label collision force $f^{i,j}_{\text{label-collision}}$ will not be applied to $label_i$. But, the opposite forces $f^{j,i}_{\text{label-collision}}$ are still applied to $label_j$. In another case, when $label_i$ is displayed and $label_j$ is hidden, $f^{i,j}_{\text{label-collision}}$ is not applied to $label_i$ and the opposite forces $f^{j,i}_{\text{label-collision}}$ are still applied to $label_j$.

### 4.2 Real-time data

The second implementation is for real-time video stream. Since we are unable to obtain the full path of the features, our hybrid optimization method needs a small modification. In the first step, we can only use the existing joint sets to calculate the label position constraints. In order to enable the labels to have a preperception to the upcoming joint set, we predict the feature motion based on the existing data by using an exponential curve prediction model [21]. As is shown in Fig. 8, the entire workflow is slightly modified from which is

**Fig. 8** A workflow of global spatiotemporal joint optimization on real-time data.

described in Section 3.1. For the existing part, we apply static optimization following Section 3.1, and the initial label positions and the position constraints of joint set ($C_1\{F_1, F_2\}$) can be calculated. For the predicted part, if a new intersection ($C_2\{F_1, F_2, F_3\}$) occurs, the static optimization of this joint set will be constrained by the maximum intersection in the existing data. The next two steps follow the flow of the first implementation, which is converting the label position constraint into force traction to guide the label movement.

## 5 Experiments

We implement the hybrid optimization method on the epidemiological data on COVID-19 in China. Figure 9 shows the visualization results of four different frames of the epidemic data. The ring-features represent provinces of China, which are placed based on their new diagnosis ($y$-axis) and the cumulative confirmed ($x$-axis). Epidemic data of 34 provinces (some provinces in Fig. 9 are hidden due to low priority) from January 21 to April 10 are included in this experiment. This figure shows effectiveness of our hybrid optimization on static label placement, in which both readability and aesthetics are maintained. The dynamic video visualization result can be found in the ESM, which shows effectiveness of our method on label movement smoothness. To verify and evaluate the effectiveness of our hybrid optimization method on smoothness preserving, we compare the results produced by the force-based label placement method [19] with and without spatiotemporal force constraint. The force-based label placement method can place labels both with smoothness and readability for dynamic data, and thus choosing this label placement method as a base can reflect the effectiveness of our spatiotemporal force constraint in these two aspects. Here we use the force-based label placement method with the parameters mentioned in Ref. [19]. To compare the performance of methods on high density and hybrid high dynamics data, the comparisons are performed on the epidemic data of China.

As shown in Fig. 10, we compare the partial layout results on the epidemic data at four different frames. In the video sequence, JiangSu first approaches and intersects HeNan, and then HeNan approaches FuJian. Basic forces without space constraint force are shown in Fig. 10(a). Due to the absence of initial

**Fig. 9** Hybrid optimization dynamic label layout on epidemiological data on COVID-19 in China.

(a) Basic forces without space constraint force

(b) Basic forces with space constraint force

**Fig. 10** Space force constraint.

position constraints and early awareness of upcoming intersection, the motion path of the label appears chaotic and the leads are too long (in order to escape overlapping), which greatly reduces the readability of labels. Oppositely, the path of the label can be planned rationally with space constraint force, and initializing the label position can also effectively improve the label movement and reduce the visual confusion caused by unreasonable paths. Complete video of the comparison experiments can be found in the ESM.

Figure 11 shows the comparison results of basic forces with or without the time constraint force. Partial features of epidemic data are displayed in this experiment. As is shown in Fig. 11, the movement velocity of HeBei is much bigger than other features.

(a) Basic forces without time constraint force

(b) Basic forces with time constraint force

**Fig. 11** Time force constraint.

In a situation that there is a huge difference in velocity between the features, the basic forces cannot timely adjust the label's motion trajectory to avoid label overlap. It is obviouse that in Fig. 11(a), with HeBei approaching quickly, the label of HeiLongJiang cannot elude the feature in time, so that the lead line has to be stretched, which seriously affects the readability. However, as shown in Fig. 11(b), the time constraint force can make the label perceive the approaching feature in advance and adjust the label position in advance, so as to make the label movement more reasonable and smooth. The comparison video also can be found in the ESM.

Figure 12 shows the performance comparison of our proposed method on real-time data stream. Two example provinces are FuJian (purple) and GuangXi (Green). The dotted line represents the movement trajectory of the province based on the real data, while the solid line represents the movement trajectory of the province based on the prediction as described in Section 4.2. The deviation of tag trajectory and the result of tag layout show that our method can carry out automatic layout of moving labels on the real-time data stream of real data.

In order to evaluate the performance of our method quantitatively, we compare the results produced by static optimization method [6], force-based method [19], and our hybrid optimization method. The static optimization method can place labels with both rationality and readability, and the force-based label placement method can maintain the smoothness of label movement. Thus we compare these labeling methods to reflect the effectiveness of our hybrid optimization in aforementioned three aspects. The comparisons are performed on epidemiological data on COVID-19 in China.

As shown in Table 1, we compared the results in the following aspects: the overlap between labels and labels and overlap between labels and features $S_{\text{Overlap}}$, the distance from the label to the corresponding feature $S_{\text{Position}}$, the number of leader line intersections $S_{\text{Aesthetics}}$, and the relative position changes of labels between adjacent frames $S_{\text{Smoothness}}$.

The first three are based on the static label layout quality rules [18], which mainly evaluates the quality of the label layout in each frame, including rationality

**Table 1** Evaluation of the effectiveness of the hybrid optimization method

| Method | Overlap | Position | Aesthetics | Smoothness | |
|---|---|---|---|---|---|
| | | | | $\theta$ | $r$ |
| Static optimization [18] | 86.6 | 147.1 | 8.2 | 61.6 | 102.5 |
| Force based [19] | 116.7 | 157.7 | 7.7 | 67.9 | 36.9 |
| Hybrid optimization | 92.2 | 156.8 | 7.9 | **47.8** | **28.2** |

**Fig. 12** Label layout for real-time data stream. Two example provinces are FuJian (purple) and GuangXi (green). The dotted line represents the movement trajectory of the city based on the real data, while the solid line represents the prediction data.

and readability. The calculation methods are similar to the calculation of energy terms described in Section 3.1.1 without adjustment weights. The calculation formulas are as follows:

$$S_{\text{Overlap}} = \frac{1}{M}\sum_{k}^{M}\left(\sum_{i}^{N}\sum_{j\neq i}^{N} O_{i,j} + \sum_{i}^{N}\sum_{j}^{N} P_{i,j}\right)$$

where $N$ is the number of features, and $M$ is the number of frames. As is shown in Fig. 2, $O_{i,j}$ is the area of overlap between label $i$ and label $j$, and $P_{i,j}$ is the area of overlap between label $i$ and feature $j$. The unit of overlap is $pixel^2$.

$$S_{\text{Position}} = \frac{1}{M}\sum_{k}^{M}\sum_{i}^{N} r_i$$

where $r_i$ is the distance between the label and feature as shown in Fig. 3(a). The unit of distance is *pixel*.

$$S_{\text{Aesthetics}} = \frac{1}{M}\sum_{k}^{M}\sum_{i}^{N} I_i$$

where $I_i$ is the number of leader line intersections.

The last item evaluates the smoothness of label movement. We calculate the relative position changes of labels between adjacent frames. In the feature-centered polar coordinate system, the relative position changes include two parts ($S^{\theta}_{\text{Smoothness}}$ and $S^{r}_{\text{Smoothness}}$). The calculation formula is as follows:

$$S^{\theta}_{\text{Smoothness}} = \frac{1}{M-1}\sum_{k}^{M-1}\sum_{i}^{N}|\theta_{i,k} - \theta_{i,k+1}|$$

where $\theta_i$ is the angle of the label in the corresponding feature-centered polar coordinate system as shown in Fig. 3(a). The unit is *degree*.

$$S^{r}_{\text{Smoothness}} = \frac{1}{M-1}\sum_{k}^{M-1}\sum_{i}^{N}|r_{i,k} - r_{i,k+1}|$$

where $r_i$ is the distance between the label and feature, and the unit is *pixel*.

According to the data in Table 1, the average overlap area and label–feature distance of static optimization method are less than the other two methods' results. Although as a trade off, the number of lead line intersections increases. The overall quality of label layouts produced by static optimization method is slightly better than those produced by force-based method. However, the static optimization method has a poor performance in the smoothness of label movement, although we add the change of label position between frames as an energy term to the objective function. Compared to static optimization method, the results produced by force-based method are much better in terms of smoothness. However, when dealing with high density and hybrid high dynamics data, the quality of label layouts produced by force-based method with basic forces becomes poor because of lacking good path planning and decoupling of high differential dynamic features. The hybrid optimization method proposed in this paper can effectively solve the confusion of label path caused by dense data intersection, obtain better initial label position through global optimization, and reasonably plan the label path through joint sets decoupling. The quality of label layouts is comparable to that produced by static optimization method. Further more, the introduced time force can effectively solve the problem of label overlap caused by the huge difference of feature speed. In terms of smoothness of label movement, hybrid optimization method is also better than the other two methods.

Three more experiments of real-world data are shown in the ESM. Additional screen shots are found in Appendix B.

## 6 Conclusions

We have proposed an effective hybrid optimization method for dynamic label layouts. The hybrid framework consists of global spatiotemporal joint optimization and dynamic label movement planning. In global spatiotemporal joint optimization, the initial label positions and position constraints of joint sets are calculated. In dynamic label movement planning, the position constraints are converted into forces to guide the label motion, and four basic forces and a temporal force are introduced to maintain the smoothness of label movement. Experiments on real-world datasets show that our hybrid optimization dynamic labeling method is able to produce clear and legible label layouts on each frame and smooth label movements in the dynamic process.

Our current hybrid optimization dynamic labeling framework needs to be improved in some respects in the future. First, the parameter settings should be optimized. We aim at designing a adaptive parameter selection method, which can greatly accelerate the process of adjusting parameters. Second, to achieve real-time labeling, we need an efficient computation of forces between labels. The GPU can be used for parallel processing of these tasks [20].

## Appendix A Parameters

These two tables presents all parameters for static optimization label layout method and the hybrid optimization dynamic label layout method.

**Table A1** Parameters for the static optimization label layout method

| Description | Symbol | Value | Unit |
|---|---|---|---|
| Area of label–label overlap | $W_{\text{label}-\text{label}}$ | 80 | — |
| Area of label–feature overlap | $W_{\text{label}-\text{feature}}$ | 50 | — |
| Orient score | $W_{\text{orient}}$ | 1–4 | — |
| Distance of label–feature | $W_{\text{distance}}$ | 20 | — |
| Out of axes | $W_{\text{out}-\text{of}-\text{axes}}$ | 320 | — |
| Number of intersecting lead lines | $W_{\text{intersect}}$ | 1 | — |

**Table A2** Parameters for the hybrid optimization dynamic label layout method

| Description | Symbol | Value | Unit |
|---|---|---|---|
| Label–label collision force scale | $w_{\text{label}-\text{collision}}$ | 40–60 | — |
| Label–label collision margin | $D_{\text{label}-\text{collision}}$ | 30 | pixel |
| Label–feature collision force scale | $w_{\text{feature}-\text{collision}}$ | 50–100 | — |
| Label–feature collision margin | $D_{\text{feature}-\text{collision}}$ | 17 | pixel |
| Pulling force scale | $w_{\text{pull}}$ | 25 | — |
| Pulling force margin | $D_{\text{pull}}$ | 18 | pixel |
| Friction force scale | $w_{\text{friction}}$ | 6 | — |
| Static friction coefficient | $c_{\text{friction}}$ | 0.7 | — |
| Space force scale | $w_{\text{space}}$ | 20 | — |
| Time force scale | $w_{\text{time}}$ | 15 | — |
| Time of the feature movement to the future | $\delta t$ | 5 | second |

## Appendix B Dataset illustrations

Below are illustrations of the dynamic datasets labeled with hybrid optimization method. All maps are attributed to Finland, © OpenStreetMap contributors, and the GIS user community. More information on openstreetmap.org/copyright.

**Fig. A1** Live air traffic above Finland from ADS-B Exchange, adsbexchange.com.

**Fig. A2** Live road traffic from the Digitraffic dataset.

TSINGHUA UNIVERSITY PRESS Springer

**Fig. A3** Labeling of a basketball game found in the APIDIS dataset.

## Acknowledgements

The authors would like to thank all reviewers for their thoughtful comments. This work was supported by the National Key Technology R&D Program (Project No. 2017YFB1002604), the National Natural Science Foundation of China (Project Nos. 61772298 and 61832016), Research Grant of Beijing Higher Institution Engineering Research Center, and Tsinghua–Tencent Joint Laboratory for Internet Innovation Technology.

**Electronic Supplementary Material** Supplementary material is available in the online version of this article at `https://doi.org/10.1007/s41095-021-0231-y`.

## Electronic supplementary material

Supplementary material, approximately 120 MB.
